# A Novel Circular RNA circITSN2 Targets the miR-218-5p/LMO7 Axis to Promote Chicken Embryonic Myoblast Proliferation and Differentiation

**DOI:** 10.3389/fcell.2021.748844

**Published:** 2021-10-06

**Authors:** Xiaoxu Shen, Yuanhang Wei, Wei Liu, Guishuang You, Shuyue Tang, Zhenyu Su, Mingxin Du, Jian He, Jing Zhao, Yongtong Tian, Yao Zhang, Menggen Ma, Qing Zhu, Huadong Yin

**Affiliations:** ^1^Farm Animal Genetic Resources Exploration and Innovation Key Laboratory of Sichuan Province, Sichuan Agricultural University, Chengdu, China; ^2^College of Animal Science and Technology, Sichuan Agricultural University, Chengdu, China; ^3^College of Resources, Sichuan Agricultural University, Chengdu, China

**Keywords:** circITSN2, CPMs, proliferation, differentiation, miR-218-5p, LMO7

## Abstract

Circular RNA (circRNA) is a class of endogenous non-coding RNAs without 5′ and 3′ ends; an increasing number of studies show that circRNA is involved in skeletal muscle development. From our previous sequencing data, the circRNAome in breast muscle of two chicken lines with a distinct rate of muscle development, which included a fast muscle growing broiler (FMGB) and a slow muscle growing layer (SMGL), we found a novel differentially expressed circRNA generated by intersectin 2 (ITSN2) gene (named circITSN2). We verified that circITSN2 is a skeletal muscle-enriched circRNA that promotes chicken primary myoblast (CPM) proliferation and differentiation. Further molecular mechanism analysis of circITSN2 in chicken myogenesis was performed, and we found circITSN2 directly targeting miR-218-5p. Besides, miR-218-5p inhibits CPM proliferation and differentiation, which is contrary to circITSN2. Commonly, circRNAs act as a miRNA sponge to alleviate the inhibition of miRNAs on mRNAs. Thus, we also identified that a downstream gene LIM domain 7 (LMO7) was inhibited by miR-218-5p, while circITSN2 could block the inhibitory effect of miR-218-5p by targeting it. Functional analysis revealed that LMO7 also accelerates CPM proliferation and differentiation, which was similar to circITSN2 but contrary to miR-218-5p. Taken together, these results suggested that circITSN2 promotes chicken embryonic skeletal muscle development *via* relieving the inhibition of miR-218-5p on LMO7. Our findings revealed a novel circITSN2/miR-218-5p/LMO7 axis in chicken embryonic skeletal muscle development, which expands our understanding of the complex muscle development regulatory network.

## Introduction

With the development of animal breeding technology in the past century, extensive meat products from domestic animals prevent hunger and malnutrition. However, large numbers of animals are raised at a high cost, and they can produce a lot of waste gases and pollutants that are harmful to the environment; the best solution is to maximize the efficiency of animal meat production (Tan and Yin, 2017). As an emerging technology, molecular breeding technology is imperative because of its potential to improve meat production efficiency of animals (Stranzinger and Went, 1996). A well-known example is the utilization of the Myostatin gene, which has a negative role on skeletal muscle development, and the gene mutation-caused loss of function of the Myostatin gene leads to more meat production (Aiello et al., 2018). In brief, understanding the molecular regulation mechanism of animal skeletal muscle development can provide the necessary theoretical basis of molecular breeding.

The growth and hypertrophy of animal skeletal muscle fibers are mainly attributed to the proliferation and differentiation of skeletal muscle progenitor cells (Dumont et al., 2015; Chal and Pourquié, 2017), which is regulated by many coding genes and non-coding RNAs (Zhao et al., 2019). Coding genes involved in myogenesis have been well-studied (Buckingham and Rigby, 2014); also, miRNAs are extremely well-established non-coding RNAs that suppress protein synthesis *via* targeting the 3′ UTR of coding genes; a few tens of miRNAs were proven to be involved in the development of skeletal myogenesis (Ge and Chen, 2011). Muscle-specific miR-133, miR-1, and miR-206 regulate muscle stem cell proliferation and differentiation *via* targeting different genes in many organisms (Horak et al., 2016). Widely expressed miR-7 inhibits chicken primary myoblast (CPM) differentiation and proliferation by targeting KLF4 (Zhang et al., 2020), and miR-7 showed the same inhibitory role in mouse C2C12 myogenesis by targeting TCF12 (Gao et al., 2021).

Competing endogenous RNAs (ceRNAs) is the maximum studied mechanism of RNA interaction, which means that non-coding RNAs competitively absorb miRNAs to alleviate the inhibitory effect of miRNAs on the expression of target coding-genes (Tay et al., 2014). As a novel class of non-coding RNAs, circRNA is an important member of the molecular regulatory network of skeletal muscle development, which was widely reported to act as miRNA sponges to perform its function (Zhang et al., 2019; Das et al., 2020). circHIPK3 promotes proliferation and differentiation of C2C12 myoblasts through the miR-7/TCF12 axis (Gao et al., 2021). CircRILPL1 promotes bovine myoblast proliferation and differentiation *via* binding miR-145 (Shen et al., 2021a). CircINSR promotes bovine myoblast proliferation and reduces its apoptosis by sponging miR-34a (Shen et al., 2020). Although some circRNAs have been shown to regulate animal myogenesis, more potential circRNAs need to be explored to expand our understanding of skeletal muscle myogenesis regulation network.

In our previous sequencing data (SRA database with accession number PRJNA516545) (Shen et al., 2019), which contains whole transcriptome data of the breast muscle of FMGB and SMGL at embryonic day 10 (E10), E13, E16, and E19, novel circRNAs that originate from chicken ITSN2 gene (circITSN2) were found to be expressed higher in FMGB than in SMGL, and were maintained at a high expression level (ranking of the circRNA expression level: 16/4,226). ITSN2 is a scaffolding protein that is conserved in diverse organisms and involved in T-cell activity (Locard-Paulet et al., 2020), oocytes development (Zhang et al., 2017), and neuronal cell function (Seifert et al., 2007). However, the potential functions of the transcripts delivered from ITSN2 gene loci in skeletal muscle development remain unknown. Therefore, in this study, we aim to explore the potential function of circITSN2 on CPM proliferation, differentiation, and the underlying mechanisms.

## Materials and Methods

### Sample Collection

All animal experiments were approved by the Animal Care Committee of Sichuan Agricultural University; the approval number is 2020102012. A total of 200 ROSS 308 broiler (a kind of FMGB) fertilized eggs (Zhengda Food Co., Ltd., Sichuan, China) and a total of 40 White Longhorn layer (a kind of SMGL) fertilized eggs (Sundaily Farm Ecological Food Co., Ltd., Sichuan, China) were incubated in an automatic incubator (Keyu Incubation Equipment Co., Ltd., Shandong, China), with a temperature at 37°C and a humidity at 60 ± 10%. For expression pattern analysis, the breast muscle of ROSS 308 broilers and White Longhorn layers at E10, E13, E16, and E19, and 10 types of tissue, namely, lung, heart, liver, spleen, kidney, breast muscle, leg muscle, brain, intestine, and adipose of 1-day old healthy ROSS 308 broilers, were collected; all samples were kept at −80°C until RNA extraction.

### siRNAs and Vectors

All of the siRNAs and miRNA mimics were synthesized by GenePharma Co., Ltd., (Shanghai, China); the sequences are listed in Supplementary Table 1. The linear sequence of circITSN2 was synthesized and inserted into pCD2.1-ciR vector to overexpress circITSN2. For dual-luciferase analysis, the linear sequence of circITSN2 that contains the wild-type (WT) and mutation-type (MT) binding site of miR-218-5p was synthesized, and the 3′UTR of LMO7 that includes the WT and MT targeting site of miR-218-5p was also synthesized, and then these synthesized sequences were cloned into psiCHECK2 vector at Tsingke Biotechnology Co., Ltd., (Chengdu, China). All vectors were verified by Sanger sequencing after construction.

### Cell Culture

The CPMs were isolated from the breast muscle of 11-day embryos of ROSS 308 broilers, which was described in a previous report (Chen et al., 2018). The hatching eggs were sterilized with 75% ethanol, the breast muscles of chicken embryo were collected, and the bones and skin were removed carefully. Then, the muscles were minced and digested in trypsin solution at 37°C for 20 min. The digestion is terminated by a growth medium (GM), which contains RPMI 1640 Medium (Gibco, Langley, United States), 10% fetal bovine serum (Gibco), and 0.2% penicillin/streptomycin (Invitrogen, Carlsbad, United States). Thereafter, the mixed system was filtered through a cell filter with 70-mm pores twice; the filtered cells were collected by centrifugation and then maintained in the GM for serial plating to remove fibroblasts. Last, the CPMs were cultured in GM at 37°C, and in a 5% CO_2_, humidified atmosphere. In addition, the differentiation medium was used to induce CPM differentiation, which contains RPMI 1640 Medium (Gibco) and 2% horse serum (Gibco). The DF-1 cells, a chicken fibroblast cell line with high transfection efficiency, which were selected for dual-luciferase assay, and were obtained from Fuheng Biology (Shanghai, China) and cultured in GM.

The transfections were performed using Lipofectamine 3000 reagent (Invitrogen) according to the manufacturer’s instruction. The medium was changed at 8 h after transfection.

### Total RNA Extraction and Quantitative Real-Time PCR (qRT-PCR)

Total RNAs were isolated from cells or tissues using RNAiso reagent (TaKaRa, Otsu, Japan). For nuclear-cytoplasmic fractionation assay, NE-PER^TM^ Nuclear and Cytoplasmic Extraction Reagents (Thermo Fisher, Carlsbad, CA, United States) were used according to the manufacturer’s instructions, and then the total RNAs from nucleus and cytoplasm were extracted using RNAiso reagent (TaKaRa), respectively. For RNase R treatment, 1 μg of mixed total RNAs was incubated with 1-unit RNase R (Epicenter Technologies, Madison, WI, United States) at 37°C for 10 min; thereafter, RNase R was inactivated by incubating at 95°C for 10 min. For mRNA and circRNA expression analysis, cDNA synthesis was performed using TransScript One-Step gDNA Removal and cDNA Synthesis SuperMix kit (TransGen, Beijing, China) according to the manufacturer’s instructions. For miRNA expression analysis, cDNA synthesis was performed using Mir-X miRNA First-Strand Synthesis Kit (TaKaRa). The qRT-PCR analysis was performed using TB Green PCR Master Mix (Takara); each sample had three independent replicates, and the 2^–ΔΔ*Ct*^ method was used to analyze the qRT-PCR data. β-actin (for circRNA and mRNA) and U6 (for miRNA) were used as internal control, and the primers are listed in Supplementary Table 2.

### Western Blot

Chicken primary myoblasts were seeded in six-well plates and cultured in GM, and the cells were induced, differentiated, and transfected when confluence reached 80–90%. After transfection for 48 h, the total proteins were extracted using Tissue or Cell Total Protein Extraction Kit (Solarbio, Beijing, China) and the protein concentration was measured by BCA Protein Assay Kit (Solarbio) according to the manufacturer’s instructions. The equivalent total proteins in different treatments were separated by SDS-PAGE and transferred onto the PVDF membrane. The membrane was blocked using QuickBlock^TM^ Blocking Buffer (Beyotime, Shanghai, China) at room temperature for 2 h. Thereafter, the membrane was incubated with primary antibodies overnight; the primary antibody includes anti-MyoG (Biorbyt, Cambridge, United Kingdom; dilute 1:1,000) and anti-β-tubulin (ZenBio, Chengdu, China; 1:2,000) and then incubated with HRP-conjugated secondary detection antibody (ZenBio; 1:2,000). Finally, the blots were detected using enhanced chemiluminescence kit (ECL) (Beyotime).

### Luciferase Assay

DF-1 cells were seeded in 48-well plates and cultured in GM, co-transfected with WT or MT reporter vector and miR-218-5p mimic or negative mimic until the confluence reached 40–50%. After 48 h for transfection, the corresponding luciferase activity of Firefly and Renilla luciferase was detected using the Luc-Pair^TM^ Duo-Luciferase HT Assay Kit (GeneCopoeia, Rockville, MD, United States) according to the manufacturer’s instructions.

### 5-Ethynyl-2′-Deoxyuridine (EdU) Assays

Chicken primary myoblasts were seeded in 96-well plates and cultured in GM, and transfected when confluence reached 40–50%. After 48 h of transfection, cells were incubated with 50 μM EdU at 37°C for 2 h and then the Cell-Light EdU Apollo567 *In Vitro* Kits (RiboBio, Guangzhou, China) were used according to the manufacturer’s protocol. Proliferating cells were stained by EdU, and the living cell nuclei were stained by Hoechst 33342.

### CCK-8 Assay

Chicken primary myoblasts were seeded in 96-well plates and cultured in GM, after 12, 24, 36, and 48 h of transfection. The approximate number of cells were detected by a Cell Counting Kit-8 kit (Multi Sciences, Hangzhou, China) according to the manufacturer’s protocol; each treatment group had eight independent replicates. The absorbance of each sample at 450 nm wavelength was measured using a Microplate reader.

### Flow Cytometry Analysis

Chicken primary myoblasts were seeded in 12-well plates, and after 36 h for transfection, cells were collected and digested with trypsin into a single-cell suspension and fixed using 75% precooled ethanol at 4°C for 24 h. Thereafter, cells were collected by centrifugation and incubated with 500 μl of PI/RNase Staining Buffer Solution (BD Biosciences, Franklin Lakes, NJ, United States) at 37°C for 15 min and then the cell cycle flow cytometry analysis was performed by a BD AccuriC6 flow cytometer (BD Biosciences).

### Immunofluorescence

Chicken primary myoblasts were seeded in 48-well plates, and the cells were induced to differentiation for 72 h after transfected by treatments. The cells were fixed in 4% formaldehyde for 30 min and permeabilized by 0.1% Triton X-100 for 20 min and then blocked by 5% goat serum (Beyotime) at room temperature for 30 min. Thereafter, the cells were incubated with primary antibody anti-MyHC (Santa Cruz; 1:250) at 4°C for 12 h and then incubated with the Rhodamine (TRITC) AffiniPure Goat Anti-Mouse IgG (ZenBio; 1:1,000) at 37°C for 1 h. Finally, cell nucleus was stained with DAPI (Beyotime; 1:50) for 5 min. Three images were randomly captured by a fluorescence microscope (Olympus, Japan). The MyHC labeled myotube area and DAPI-labeled nuclear area (Internal control) were measured by Image-Pro Plus software, and the ratio of myotube area to nuclear area is taken as the relative myotube area.

### Bioinformatic Analysis

The circITSN2 target miRNA analysis was performed using RNAhybrid software^1^, and the miR-218-5p target genes were scanned from TargetScan website^2^ and miRDB website^3^. The dynamic trend analysis of the genes and miRNAs was performed using OmicShare website^4^. Venn analysis was performed using Draw Venn Diagram website^5^.

### Statistical Analysis

Data were presented as least squares means ± standard error of the mean (SEM) and statistical analysis was performed using SPSS 20.0 software (SPSS Inc., United States). The significant difference between means was analyzed using unpaired Student’s *t*-test and one-way ANOVA. *p* < 0.05 was considered to indicate statistical significance, *p* < 0.05 (^∗^), *p* < 0.05 (a, b), *p* < 0.01 (^∗∗^).

## Results

### Circular RNA circITSN2 Differentially Expressed During Chicken Embryonic Skeletal Muscle Development

**FIGURE 1:**
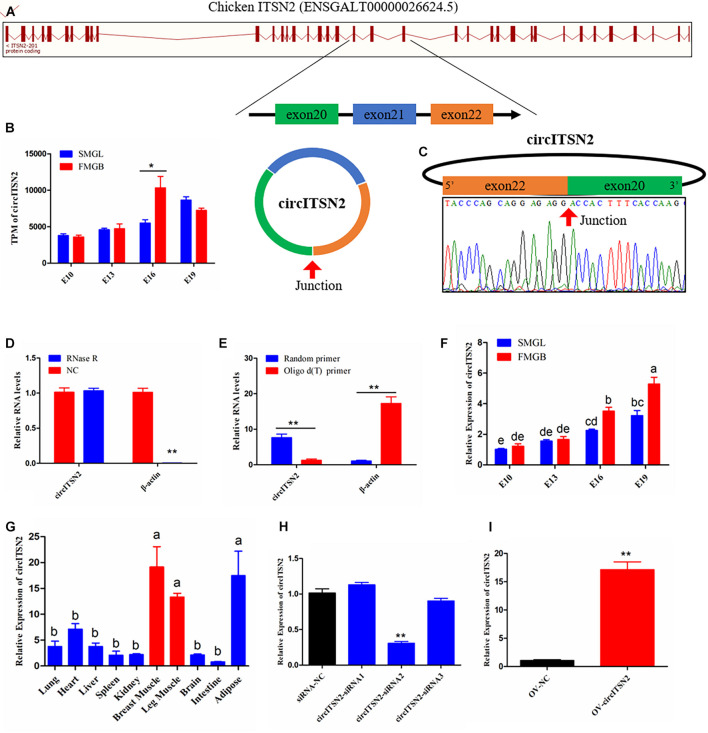
The origin, expression pattern, and characteristics of circITSN2. **(A)** Schematic diagram of circITSN2 generation. **(B)** The expression of circITSN2 in the sequencing results (TPM: transcripts per million). **(C)** The junction site of circITSN2 was detected by Sanger sequencing. **(D)** The expression level of circITSN2 and β-actin in CPMs with or without RNase R treatment. **(E)** The expression of circITSN2 and β-actin in the cDNA of CPMs generated using different reverse transcription primers. **(F)** The expression of circITSN2 in the muscle of two chicken lines with four different embryonic stages detected by qRT-PCR. **(G)** The expression pattern of circITSN2 in 10 different chicken tissues. **(H)** The expression of circITSN2 in CPMs transfected with unique siRNAs. **(I)** The expression of circITSN2 in CPMs transfected with circITSN2 overexpression vector or empty vector. Data are presented as mean ± SEM for at least three individuals; ^*ab*^*p* < 0.05, *p < 0.05, and **p < 0.01.

Among the sequencing data, a total of 10 circRNAs generated from chicken ITSN2 gene were found (Supplementary Table 3), but only the one formed by exons 20–22 (ENSGALT00000026624.5) was differentially expressed between FMGB and SMGL (*p* < 0.05; Figures 1A,B); it also represented the highest expression level in these 10 circRNAs, which we named circITSN2 (Supplementary Table 3). In order to detect the circular structure of circITSN2, the reverse splicing site of circITSN2 was amplified by a divergent primer pair and detected by Sanger sequencing; the result confirmed the junction sequence of circITSN2 (Figure 1C). RNase R resistance test showed that circular ITSN2 RNA is more resistant to the digestion of RNase R than linear β-actin mRNA (*p* < 0.01; Figure 1D); furthermore, the content of circITSN2 in the random primer N9 reverse transcription system was much higher than that in oligo d(T) primer reverse transcription system (*p* < 0.01; Figure 1E), which indicated that circITSN2 is a stable circular RNA without 3′ poly (A) tail. qRT-PCR analysis was used to determine the expression of circITSN2 in FMGB and SMGL; results showed that circITSN2 was significantly upregulated in FMGB (*p* < 0.05; Figure 1F). Besides, the expression of circITSN2 increased gradually during embryonic muscle development (*p* < 0.05; Figure 1F). Expression pattern analysis indicated that the expression of circITSN2 in skeletal muscles was far higher than other tissues (*p* < 0.05; Figure 1G). In addition, we also detected the expression pattern of linear ITSN2 mRNA; results showed that linear ITSN2 mRNA did not differentially express between FMGB and SMGL, also without a muscle-specific expression pattern (Supplementary Figures 1A,B). Altogether, these findings indicated that circITSN2 is a stable skeletal muscle-enriched circRNA, which has the potential to regulate muscle development.

We used siRNAs to investigate the effect of silencing circITSN2 on proliferation and differentiation of CPMs; three siRNAs were designed, covering the junction site of circITSN2, and among these siRNAs, siRNA2 extended a significant silencing efficiency of circITSN2 compared with siRNA negative control (NC), which was selected for further experiments (designated si-circITSN2) (*p* < 0.01; Figure 1H). Besides, an exogenous vector was used to overexpress circITSN2 in CPMs, and results showed that the overexpression vector significantly increased the expression of circITSN2 in CPMs (*p* < 0.01; Figure 1I). In addition, these siRNAs and the overexpression vector of circITSN2 did not affect the expression of linear ITSN2 mRNA (Supplementary Figures 1C,D).

### CircITSN2 Boosts CPM Proliferation

**FIGURE 2:**
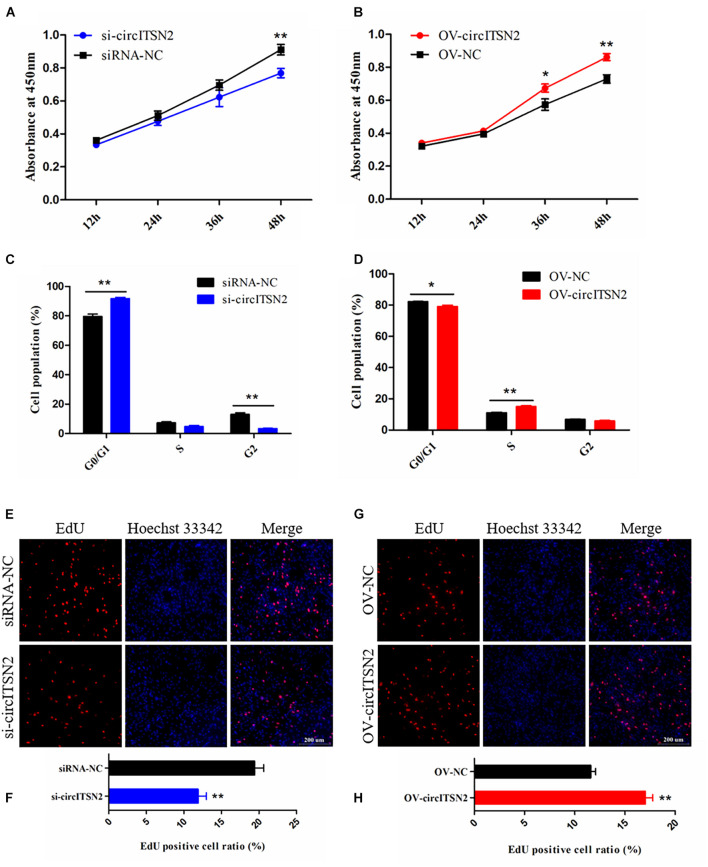
CircITSN2 promotes the proliferation of CPMs. **(A)** The growth curves of CPMs were measured by CCK-8 reagent after inhibition of circITSN2. **(B)** The growth curves of CPMs were measured by CCK-8 reagent after overexpression of circITSN2. **(C)** The cell cycle analysis of CPMs after inhibition of circITSN2. **(D)** The cell cycle analysis of CPMs after overexpression of circITSN2. **(E)** Fluorescence microscope images of proliferating CPMs detected by EdU kit after inhibition of circITSN2. **(F)** The proliferating rate of CPMs after inhibition of circITSN2. **(G)** Fluorescence microscope images of proliferating CPMs detected by EdU kit after overexpression of circITSN2. **(H)** The proliferating rate of CPMs after overexpression of circITSN2. Data are presented as mean ± SEM for at least three individuals; *p < 0.05 and **p < 0.01.

To explore the role of circITSN2 in CPM proliferation, CCK-8 assay, EdU analysis, and flow cytometry analysis were performed in CPMs after modulation of circITSN2 expression. The results of CCK-8 assay showed that knockdown of circITSN2 significantly reduced the proliferation vitality of CPMs (*p* < 0.01; Figure 2A), while the proliferation vitality was induced by circITSN2 overexpression (*p* < 0.05; Figure 2B). Flow cytometry analysis revealed that knockdown of circITSN2 suspend CPMs at the G0/G1 phase (*p* < 0.01; Figure 2C), whereas overexpression of circITSN2 promoted cells to enter the S phase and G2/M phase (*p* < 0.05; Figure 2D). Similarly, EdU analysis showed that proliferating CPMs were significantly decreased following circITSN2 knockdown (*p* < 0.01; Figures 2E,F) but increased following circITSN2 overexpression (*p* < 0.01; Figures 2G,H). Together, these findings suggested that circITSN2 promotes CPM proliferation.

### CircITSN2 Promotes CPM Differentiation

**FIGURE 3:**
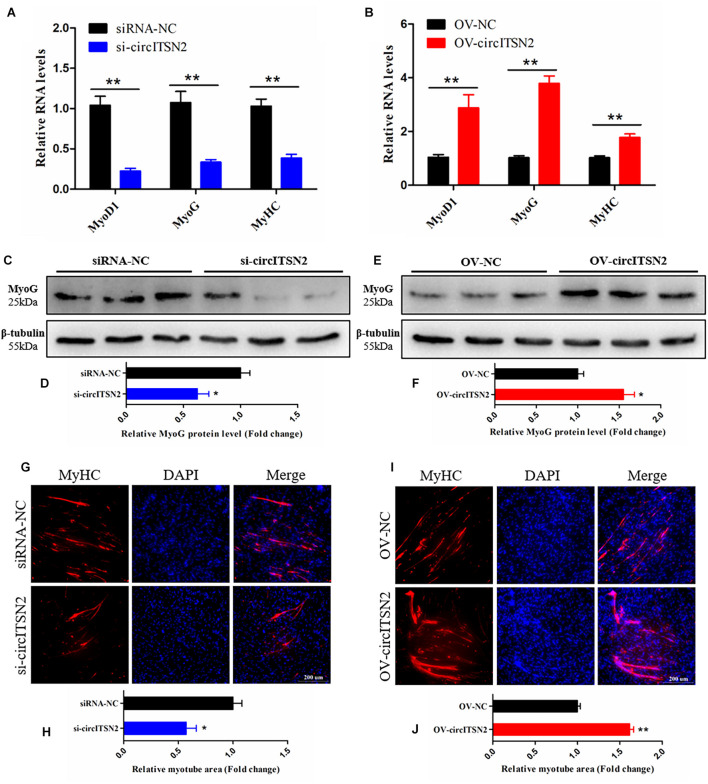
CircITSN2 promotes the differentiation of CPMs. **(A)** The relative RNA level of muscle cell differentiation marker genes in CPMs was detected by qRT-PCR after inhibition of circITSN2. **(B)** The relative RNA level of muscle cell differentiation marker genes in CPMs was detected by qRT-PCR after overexpression of circITSN2. **(C,D)** The relative protein level of MyoG in CPMs was detected by Western blot after inhibition of circITSN2. **(E,F)** The relative protein level of MyoG in CPMs was detected by Western blot after overexpression of circITSN2. **(G)** Fluorescence microscope images of MyHC staining CPMs after inhibition of circITSN2. **(H)** The relative myotube area of CPMs after inhibition of circITSN2. **(I)** Fluorescence microscope images of MyHC staining CPMs after overexpression of circITSN2. **(J)** The relative myotube area of CPMs after overexpression of circITSN2. Data are presented as mean ± SEM for at least three individuals; *p < 0.05 and **p < 0.01.

To investigate the effect of circITSN2 on CPM differentiation, qRT-PCR analysis, Western blot assay, and immunofluorescence analysis were performed in CPMs after modulation of circITSN2 expression. qRT-PCR analysis showed that the mRNA levels of three skeletal muscle differentiation key genes Myogenin (MyoG), Myogenic Differentiation 1 (MyoD1), and Myosin Heavy Chain (MyHC) were decreased by circITSN2 knockdown (*p* < 0.01; Figure 3A) while they were increased following circITSN2 overexpression (*p* < 0.01; Figure 3B). Uniformly, the protein level of MyoG showed similar changes to that of MyoG mRNA level (*p* < 0.05; Figures 3C–F). In addition, the relative myotube area stained by MyHC antibody was reduced following circITSN2 knockdown (*p* < 0.05; Figures 3G,H), whereas it was increased following circITSN2 overexpression (*p* < 0.01; Figures 3I,J). These results indicated that circITSN2 promotes CPM differentiation.

### CircITSN2 Acts as a miRNA Sponge of miR-218-5p

**FIGURE 4:**
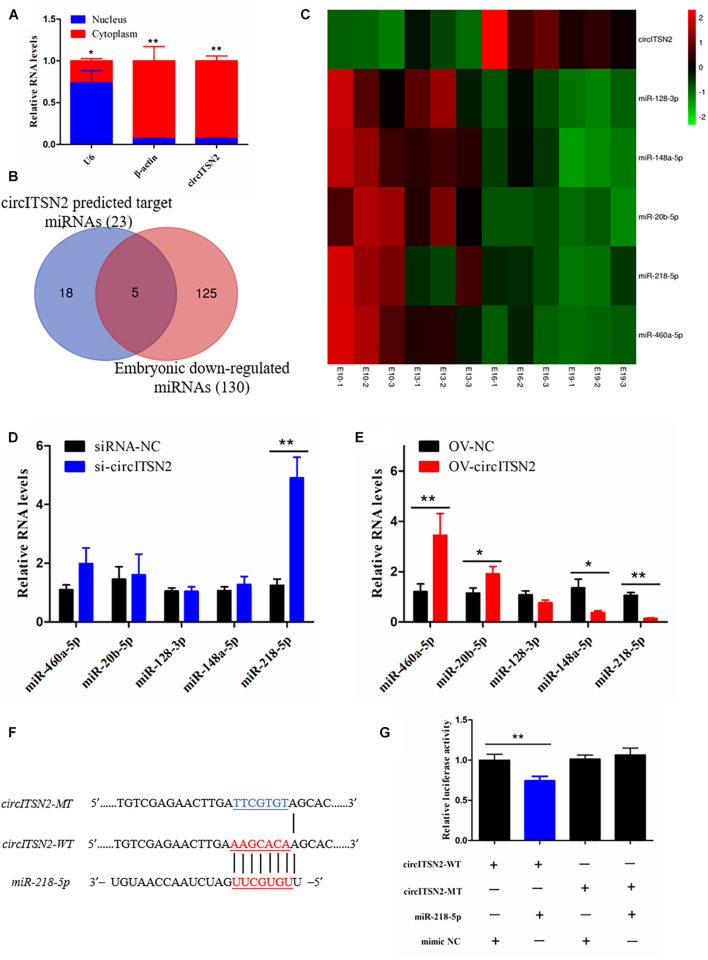
Target-miRNA scanning revealed circITSN2 directly targets miR-218-5p in CPMs. **(A)** The expression level of circITSN2 in the cytoplasm (red) and nuclei (blue) of CPMs. **(B)** The Venn analysis of circITSN2 predicted target miRNAs and embryonic downregulated miRNAs. **(C)** The sequencing expression level of five potential targeted miRNAs of circITSN2. **(D)** The expression level of five potential targeted miRNAs of circITSN2 in CPMs after inhibition of circITSN2. **(E)** The expression level of five potential targeted miRNAs of circITSN2 in CPMs after overexpression of circITSN2. **(F)** Dual-luciferase reporter vectors contain wild type or mutant type of the target site of miR-218-5p on circITSN2. **(G)** The relative luciferase activity in DF-1 cells was detected after co-transfecting with the dual-luciferase reporter vectors and miRNA mimics. Data are presented as mean ± SEM for at least three individuals; *p < 0.05 and **p < 0.01.

To explore the molecular mechanism in which circITSN2 regulated CPM proliferation and differentiation, we first detected its subcellular localization, and results showed that circITSN2 was significantly enriched in the cytoplasm (*p* < 0.05; Figure 4A). It is noteworthy that an increasing number of studies showed that circRNAs adsorb miRNAs in the cytoplasm to regulate downstream target genes (Das et al., 2020). Thus, we predicted the potential target miRNAs using RNAhybrid software, 23 miRNAs were found may be regulated by circITSN2 (Supplementary Table 4). Previous reports showed that circRNAs and miRNAs in ceRNA regulatory network have opposite expression patterns (Ouyang et al., 2018a; Shen et al., 2021b), and circITSN2 gradually increased during embryonic muscle development. Besides, previously we sequenced the miRNAs of the breast muscle of embryonic chicks at E10, E13, E16, and E19 (SRA database with accession number PRJNA516545). We re-scanned the sequencing data using Expression trend analysis (Supplementary Figure 2); 130 miRNAs were revealed to be downregulated during embryonic muscle development (Supplementary Table 5); further Venn analysis found that five target miRNAs of circITSN2 decreased gradually over the embryonic muscle development (Figures 4B,C), which suggested that these miRNAs are more likely to be regulated by circITSN2.

Then, in order to find miRNAs directly regulated by circITSN2, qRT-PCR analysis was performed and results showed that knockdown circITSN2 upregulated the expression of miR-218-5p (*p* < 0.01; Figure 4D) and overexpression of circITSN2 upregulated the level of miR-460a-5p and miR-20a-5p and downregulated the level of miR-218-5p and miR-148a-5p (*p* < 0.05; Figure 4E). Among these miRNAs, miR-218-5p is the most obvious miRNA regulated by circITSN2. To confirm the combination of circITSN2 and miR-218-5p, dual-luciferase reporter vectors including the fragment of circITSN2 that contains WT and MT miR-218-5p binding site were constructed and co-transfected with miR-218-5p mimic or mimic NC into DF-1 cells (Figure 4F). Results showed that miR-218-5p significantly reduced the relative luciferase activity of WT vector, but had no effect on that of MT vector (*p* < 0.01; Figure 4G). Taken together, these results confirmed that circITSN2 can act as a miRNA sponge of miR-218-5p.

### CircITSN2 Eliminates the Inhibition Effect of miR-218-5p on CPM Proliferation and Differentiation

**FIGURE 5:**
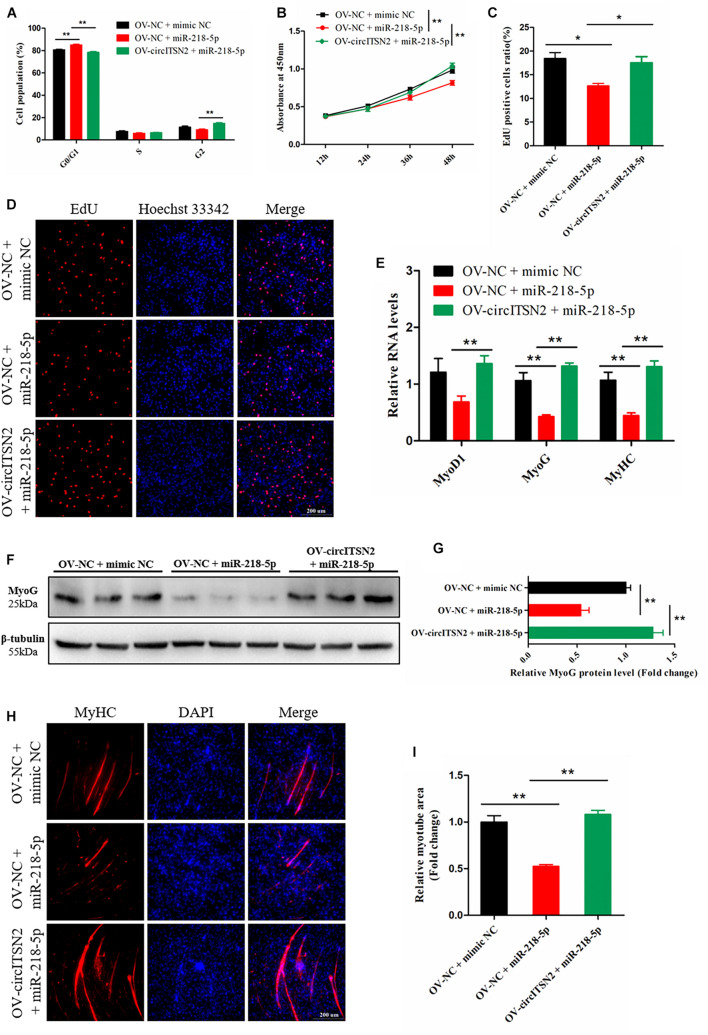
CircITSN2 eliminates the inhibition effect of miR-218-5p on CPM proliferation and differentiation. **(A)** The cell cycle analysis of CPMs that overexpressed miR-218-5p or co-overexpressed miR-218-5p and circITSN2. **(B)** The growth curves of CPMs that overexpressed miR-218-5p or co-overexpressed miR-218-5p and circITSN2 were measured by CCK-8 reagent. **(C)** The proliferating rate of CPMs that overexpressed miR-218-5p or co-overexpressed miR-218-5p and circITSN2. **(D)** Fluorescence microscope images of proliferating CPMs that overexpressed miR-218-5p or co-overexpressed miR-218-5p and circITSN2 were detected by EdU analysis kit. **(E)** The relative RNA level of muscle cell differentiation marker genes in CPMs that overexpressed miR-218-5p or co-overexpressed miR-218-5p and circITSN2 was detected by qRT-PCR. **(F,G)** The relative protein level of MyoG in CPMs that overexpressed miR-218-5p or co-overexpressed miR-218-5p and circITSN2 was detected by Western blot. **(H)** Fluorescence microscope images of MyHC staining CPMs that overexpressed miR-218-5p or co-overexpressed miR-218-5p and circITSN2. **(I)** The relative myotube area of CPMs that overexpressed miR-218-5p or co-overexpressed miR-218-5p and circITSN2. Data are presented as mean ± SEM for at least three individuals; *p < 0.05 and **p < 0.01.

To determine the relationship between circITSN2 and miR-218-5p on CPM proliferation, CCK-8 assay, EdU analysis, and flow cytometry analysis were performed in CPMs that overexpressed miR-218-5p or co-overexpressed miR-218-5p and circITSN2. CPMs that overexpressed miR-218-5p increased the number of G0/G1 phase cells compared to the control group (*p* < 0.01; Figure 5A). Likewise, CCK-8 assay showed that overexpressed miR-218-5p decreased the proliferate vitality of CPMs (*p* < 0.01; Figure 5B) and also decreased the proliferating CPMs in EdU analysis results (*p* < 0.05; Figures 5C,D). These findings indicated that miR-218-5p inhibits CPM proliferation. Interestingly, the inhibition effect of miR-218-5p was impaired when co-overexpressed with circITSN2 simultaneously (Figures 5A–D).

To determine the relationship between circITSN2 and miR-218-5p on CPM differentiation, qRT-PCR analysis, Western blot assay, and immunofluorescence analysis were performed in CPMs after overexpression of miR-218-5p or co-overexpression of miR-218-5p and circITSN2. Overexpression of miR-218-5p significantly decreased the mRNA level (*p* < 0.01; Figure 5E) and protein level (*p* < 0.01; Figures 5F,G) of muscle cell differentiation marker genes, and the relative myotube area was reduced by the excessive expression of miR-218-5p (*p* < 0.01; Figures 5H,I). These results suggested that miR-218-5p inhibits CPM differentiation; however, the suppression effect of miR-218-5p was eliminated by co-overexpressing with circITSN2 (Figures 5E–I).

### CircITSN2 Regulates LMO7 via Targeting miR-218-5p in CPMs

**FIGURE 6:**
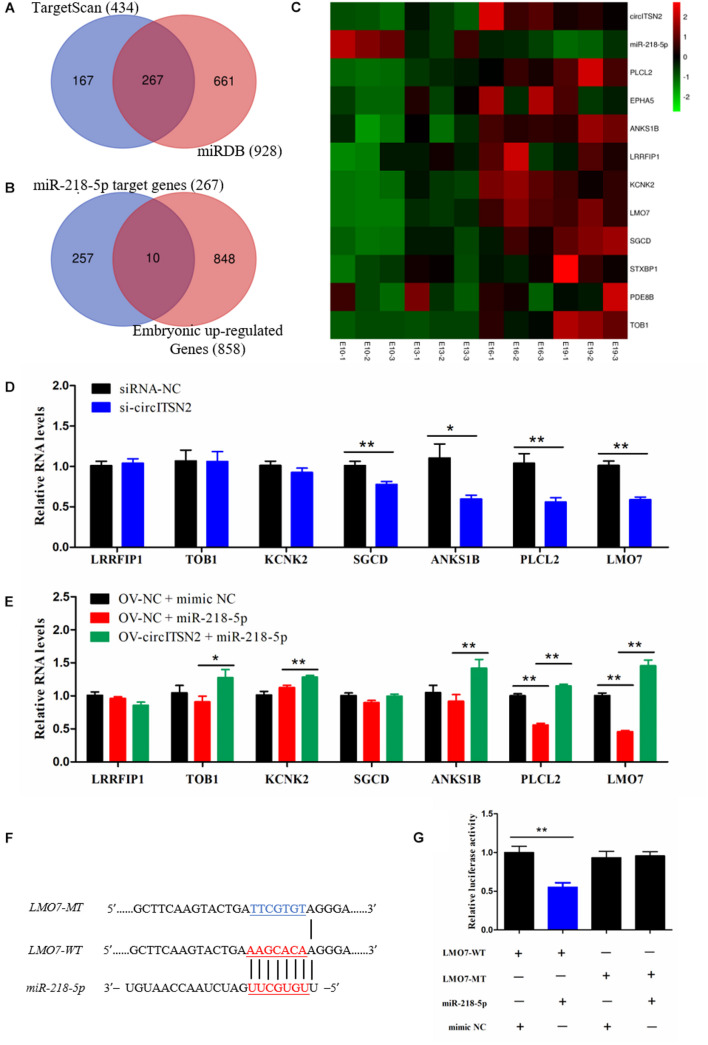
CircITSN2 regulates LMO7 *via* targeting miR-218-5p in CPMs. **(A)** The Venn analysis of miR-218-5p target genes from the TargetScan website and miRDB website. **(B)** The Venn analysis of miR-218-5p target genes and the embryonic upregulated genes. **(C)** The sequencing expression level of 10 potential targeted genes of circITSN2/miR-218-5p axis. **(D)** The expression level of potential targeted genes of the circITSN2/miR-218-5p axis in CPMs after inhibition of circITSN2. **(E)** The expression level of potential targeted genes of circITSN2/miR-218-5p axis in CPMs that overexpressed miR-218-5p or co-overexpressed miR-218-5p and circITSN2. **(F)** Dual-luciferase reporter vectors contain wild type or mutant type of the target site of miR-218-5p on LMO7. **(G)** The relative luciferase activity in DF-1 cells was detected after co-transfected with the dual-luciferase reporter vectors and miRNA mimics. Data are presented as mean ± SEM for at least three individuals; *p < 0.05 and **p < 0.01.

Usually, in the ceRNA mechanism, circRNAs regulate protein translation *via* block the inhibition effect of miRNAs on mRNAs (Tay et al., 2014). Thus, to identify which genes were regulated by the circITSN2/miR-218-5p axis, we first predicted the target genes of miR-218-5p by searching TargetScan and miRDB websites (Supplementary Table 6); Venn analysis revealed 267 target genes in the intersection of two software that predicted results (Figure 6A), and these genes were selected for subsequent analysis. In addition, we found that the expression of circITSN2 was upregulated whereas the expression of miR-218-5p was downregulated during embryonic muscle development mentioned above (Figure 4C). Therefore, the genes upregulated during embryonic muscle development are the theoretical circITSN2-regulated candidates. Hence, expression trend analysis of our previous sequencing data (SRA database with accession number PRJNA516545) showed that 858 genes were gradually increased during embryonic muscle development (Supplementary Figure 3 and Supplementary Table 7). Finally, Venn analysis showed only 10 theoretical circITSN2/miR-218-5p axis-regulated candidates were upregulated during embryonic muscle development (Figures 6B,C).

To investigate which of these 10 theoretical circITSN2-regulated candidates is actually regulated by the circITSN2/miR-218-5p axis, qRT-PCR analysis was performed in CPMs after inhibition or overexpression of circITSN2, with the exception of three genes Syntaxin binding protein 1 (STXBP1), Phosphodiesterase 8B (PDE8B), and EPH receptor A5 (EPHA5) with low expression in CPMs that could not be accurately detected. Results showed that knockdown of circITSN2 decreased the expression of Sarcoglycan delta (SGCD), Ankyrin repeat and sterile alpha motif domain containing 1B (ANKS1B), Phospholipase C-like 2 (PLCL2), and LMO7 (*p* < 0.05; Figure 6D), overexpression of miR-218-5p significantly reduced the expression of PLCL2 and LMO7 (*p* < 0.01; Figure 6E), and co-overexpression of circITSN2 and miR-218-5p rescued the expression of Transducer of ERBB2, 1 (TOB1), Potassium two pore domain channel subfamily K member 2 (KCNK2), PLCL2, and LMO7 compared with overexpressed miR-218-5p alone (*p* < 0.05; Figure 6E). Among these results, we noticed that LMO7 was the most significant gene that was regulated by both circITSN2 and miR-218-5p. To further identify the targeting relationship between LMO7 and miR-218-5p, dual-luciferase reporter vectors were constructed and transfected into DF-1 cells (Figure 6F), and results confirmed that miR-218-5p directly targets LMO7 (*p* < 0.01; Figure 6G). In short, our results indicated that circITSN2 regulates LMO7 *via* targeting miR-218-5p in CPMs.

### Knockdown of LMO7 Inhibits the Proliferation and Differentiation of CPMs

**FIGURE 7:**
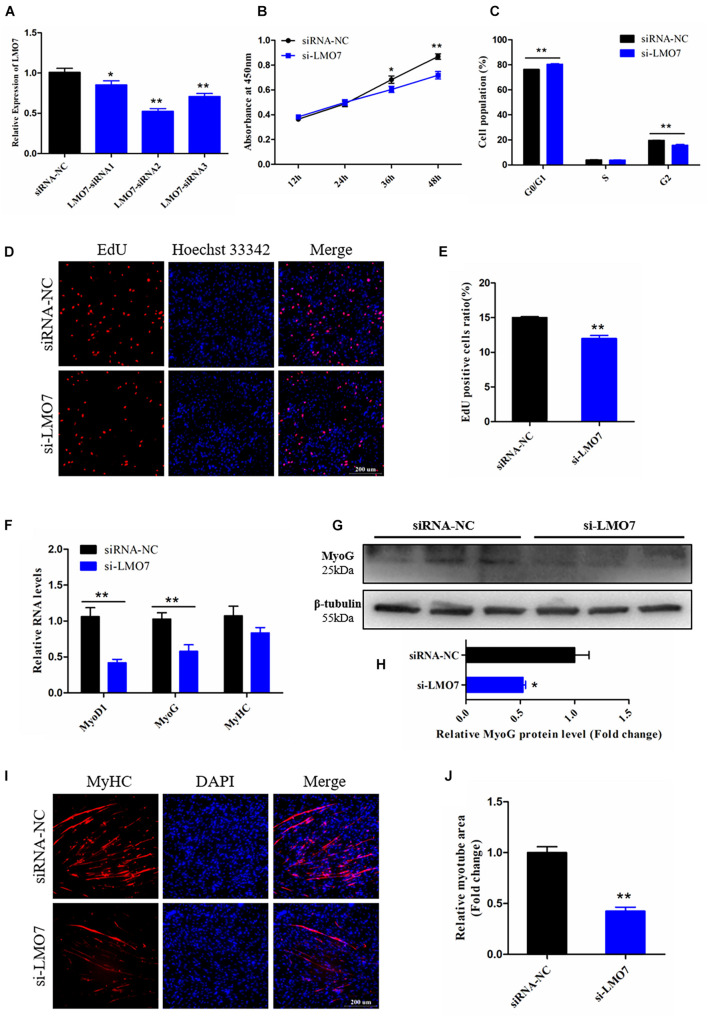
Knockdown of LMO7 inhibits the proliferation and differentiation of CPMs. **(A)** The expression of LMO7 in CPMs transfected with unique siRNAs. **(B)** The growth curves of CPMs were measured by CCK-8 reagent after inhibition of LMO7. **(C)** The cell cycle analysis of CPMs after inhibition of LMO7. **(D)** Fluorescence microscope images of proliferating CPMs detected by the EdU kit after inhibition of LMO7. **(E)** The proliferating rate of CPMs after inhibition of LMO7. **(F)** The relative RNA level of muscle cell differentiation marker genes in CPMs was detected by qRT-PCR after inhibition of LMO7. **(G,H)** The relative protein level of MyoG in CPMs was detected by Western blot after inhibition of LMO7. **(I)** Fluorescence microscope images of MyHC staining CPMs after inhibition of LMO7. **(J)** The relative myotube area of CPMs after inhibition of LMO7. Data are presented as mean ± SEM for at least three individuals; *p < 0.05 and **p < 0.01.

We designed and synthesized three corresponding siRNAs of LMO7 to modify its expression in CPMs, and then qRT-PCR analysis results showed that LMO7 were successfully silenced in CPMs, and the siRNA2 with the best silence effect was chosen for the following experiments (designated si-LMO7) (*p* < 0.01; Figure 7A).

To verify the potential function of LMO7 on CPM proliferation, several cell proliferation detection techniques were performed in the LMO7 knockdown CPMs. CCK-8 assay showed that the decreased expression of LMO7 resulted in the decrease of cell proliferate vitality (*p* < 0.05; Figure 7B), which also significantly repressed cell transition from the G1 phase to S phase (*p* < 0.01; Figure 7C). EdU analysis showed a similar result to the CCK-8 assay, revealing that knockdown of LMO7 decreased the number of proliferating CPMs (*p* < 0.01; Figures 7D,E). These results illustrated that LMO7 may play positive roles in CPM proliferation.

To identify the involvement of LMO7 in CPM differentiation. Skeletal muscle cell differentiation key genes were detected and results showed that the mRNA levels of these genes were decreased following LMO7 knockdown (*p* < 0.01; Figure 7F), and the protein level of MyoG was also reduced by knockdown of LMO7 (*p* < 0.05; Figures 7G,H). In addition, knockdown of LMO7 significantly inhibits the myotube formation of CPMs (*p* < 0.01; Figures 7I,J). Thus, these results lead us to believe that LMO7 has a positive function in CPM differentiation.

## Discussion

Skeletal muscle is one of the most important tissues for animals, which regulates the locomotion and metabolism of the animal body (Blaauw et al., 2013). For domestic animals, skeletal muscle development is associated with specific differences in the quality and quantity of the meat (Picard et al., 2010). The process of myogenesis is precisely managed by many factors including many genes (Buckingham and Rigby, 2014). LMO7, a muscle growth-associated gene, upregulates the expression of MyoD and Myf5 by targeting their promoter to boost C2C12 myogenic differentiation (Dedeic et al., 2011). On the other hand, LMO7 is required for emerin gene transcription, which is essential for muscle regeneration *via* Rb-MyoD pathways (Bakay et al., 2006; Holaska et al., 2006). In addition, knockdown of LMO7 decreased the number of MyoD-positive myoblasts and inhibited myotube formation, which suggested that LMO7 plays an important role in chicken myoblast survival and differentiation (Possidonio et al., 2016). Coincidentally, in the present study, our data also proved that the silence of LMO7 blocked cell cycle progression and inhibited the proliferative vitality of CPMs as well as decreased the expression level of myogenic differentiation marker genes and restrained the formation of myotubes. Our results provide a similar inference to the previous studies, and LMO7 plays a stimulative role in skeletal muscle development. As for the potential mechanisms of LMO7 regulating chicken myogenesis, more research is needed.

The muscle-related coding genes could be directly regulated by miRNAs, except for several muscle-specific miRNAs, and some widely expressed miRNAs also showed involvement in myogenesis. MiR-218-5p is a widely expressed miRNA that exerts an inhibitory function and promoted cell migration *via* targeting PDGFRα during cardiomyocyte differentiation in mouse (Xu et al., 2019). In zebrafish, the false pattern of miR-218 may cause cardiac malformation (Chiavacci et al., 2012). Although miR-218-5p was widely reported to be involved in the development of myocardium, the potential role in skeletal muscle remains unclear. A report showed that reducing the expression of miR-218-5p could alleviate the myotonic dystrophy phenotypes *via* upregulated MBNLs (Cerro-Herreros et al., 2018). In our study, we found that the excessive expression of miR-218-5p diminished the multiplication and differentiation of CPMs. Combined with previous studies, there are grounds to believe that miR-218-5p is a restriction factor of skeletal muscle development. Furthermore, we also found that miR-218-5p directly targets LMO7, which attenuated the expression and may block the promotion effect of LMO7 on chicken myogenesis. Our results not only demonstrated the role of miR-218-5p on chicken muscle development but also verified the target regulating relationship between miR-218-5p and LMO7.

CircRNAs were found to be widely expressed in many tissues, and recent studies also indicated that circRNAs play roles in muscle growth (Ge and Chen, 2011). CircLMO7 was highly expressed in embryo bovine muscle compared to adult and promoted bovine myoblast survival by sponging miR-378a-3p (Wei et al., 2017). CircRBFOX2 was differentially expressed during Xinghua chicken embryonic muscle development and promoted CPM proliferation by targeting miR-1 and miR-206 (Ouyang et al., 2018b). Our previous report revealed hundreds of differentially expressed circRNAs between FMGB and SMGL, and a SMGL-enriched circRNA circTMTC1 inhibits chicken skeletal muscle satellite cell differentiation by targeting miR-128-3p (Shen et al., 2019). In this study, a novel circular ITSN2 RNA was found highly expressed in FMGB, and functional analysis indicated that circITSN2 enhanced the proliferation and differentiation of CPMs; our data suggest the positive role of circITSN2 in chicken muscle development, which is diametrically opposed to miR-218-5p. Furthermore, we revealed the adsorption relationship between circITSN2 and miR-218-5p. It is reported that miR-218-5p could be absorbed and regulated by many circRNAs, like circEIF4G2 (Lin et al., 2020; Xu et al., 2020) and circSAMD4A (Wei et al., 2020). Our research also identified a novel upstream circRNA sponge for miR-218-5p in chicken muscle development.

Embryonic development is a very complicated biological process, including the formation of many organizations (Tavian and Péault, 2005; Yang, 2009; Rubinstein et al., 2016). It is reported that a certain number of muscle fibers formed in embryo, which will not increase after birth; this proves the importance of embryonic muscle development (Wigmore and Stickland, 1983; Buckingham et al., 2003; Yan et al., 2013). We found that a novel circRNA circITSN2 gradually increased with the development of embryonic skeletal muscle, and it was found to play a positive role in myogenesis; we speculate that it may promote the formation of skeletal muscle fibers in embryonic chicks. Through the expression pattern-based target miRNA/mRNA scanning, we revealed that a novel circITSN2/miR-218-5p/LMO7 axis was involved in embryonic myogenesis, which provides a novel insight into embryonic muscle development. Furthermore, the genomic variations affecting the circITSN2/miR-218-5p/LMO7 axis may be potential targets used for molecular breeding to improve local chicken meat yield.

**FIGURE 8:**
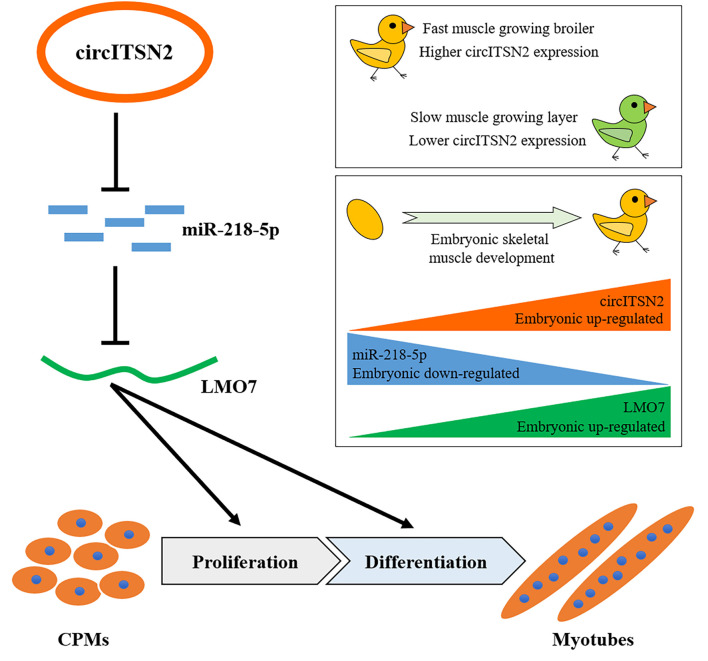
Schematic model of the circITSN2/miR-218-5p/LMO7 axis in chicken embryonic skeletal muscle development.

In conclusion, a novel circITSN2/miR-218-5p/LMO7 axis was demonstrated in chicken embryonic muscle development, and their corresponding functions in CPM proliferation and differentiation have also been verified. In other words, circITSN2 promotes chicken myogenesis *via* alleviating the inhibition of miR-218-5p on LMO7 (Figure 8).

## Data Availability Statement

The original contributions presented in the study are included in the article/Supplementary Material, further inquiries can be directed to the corresponding author/s.

## Ethics Statement

The animal study was reviewed and approved by Animal Care Committee of Sichuan agricultural university.

## Author Contributions

XS, YW, WL, and HY: conceptualization. XS, YW, GY, and ST: formal analysis. HY and QZ: funding acquisition. XS, YW, WL, ST, ZS, MD, JH, JZ, and YT: investigation. XS: methodology. YZ: project administration. XS, YW, and WL: writing—original draft. MM, QZ, and HY: writing—review and editing. All authors contributed to the article and approved the submitted version.

## Conflict of Interest

The authors declare that the research was conducted in the absence of any commercial or financial relationships that could be construed as a potential conflict of interest.

## Publisher’s Note

All claims expressed in this article are solely those of the authors and do not necessarily represent those of their affiliated organizations, or those of the publisher, the editors and the reviewers. Any product that may be evaluated in this article, or claim that may be made by its manufacturer, is not guaranteed or endorsed by the publisher.

## Footnotes

1
https://bibiserv.cebitec.uni-bielefeld.de/rnahybrid/

2
http://www.targetscan.org/vert_72/

3
http://mirdb.org/

4
https://www.omicshare.com/tools/

5
http://bioinformatics.psb.ugent.be/webtools/Venn/
