# Supplementary material for: A Novel Circular RNA circITSN2 Targets the miR-218-5p/LMO7 Axis to Promote Chicken Embryonic Myoblast Proliferation and Differentiation
Source: Front Cell Dev Biol. 2021 Oct 6;9:748844. doi: 10.3389/fcell.2021.748844 (PMC8526564; doi:10.3389/fcell.2021.748844)
Supplement: Supplementary file 1 [file Data_Sheet_1.docx]

**A novel circular RNA circITSN2 targets the miR-218-5p/LMO7 axis to promote chicken embryonic myoblast proliferation and differentiation**

Xiaoxu Shen^1^, Yuanhang Wei^1^, Wei Liu^2^, Guishuang You^1^, Shuyue Tang^1^, Zhenyu Su^2^, Mingxin Du^2^, Jian He^2^, Jing Zhao^1^, Yongtong Tian^1^, Yao Zhang^1^, Menggen Ma^3^, Qing Zhu^1*^ and Huadong Yin^1*^

^1^ Farm Animal Genetic Resources Exploration and Innovation Key Laboratory of Sichuan Province, Sichuan Agricultural University, Chengdu 611130, China.

^2^ College of Animal Science and Technology, Sichuan Agricultural University, Chengdu 611130, China.

^3^ College of Resources, Sichuan Agricultural University, Chengdu 611130, China.

*** Corresponding author:**

Prof. Qing Zhu; E-mail: zhuqing@sicau.edu.cn.

Prof. Huadong Yin; E-mail: yinhuadong@sicau.edu.cn.

**Supplementary figures**


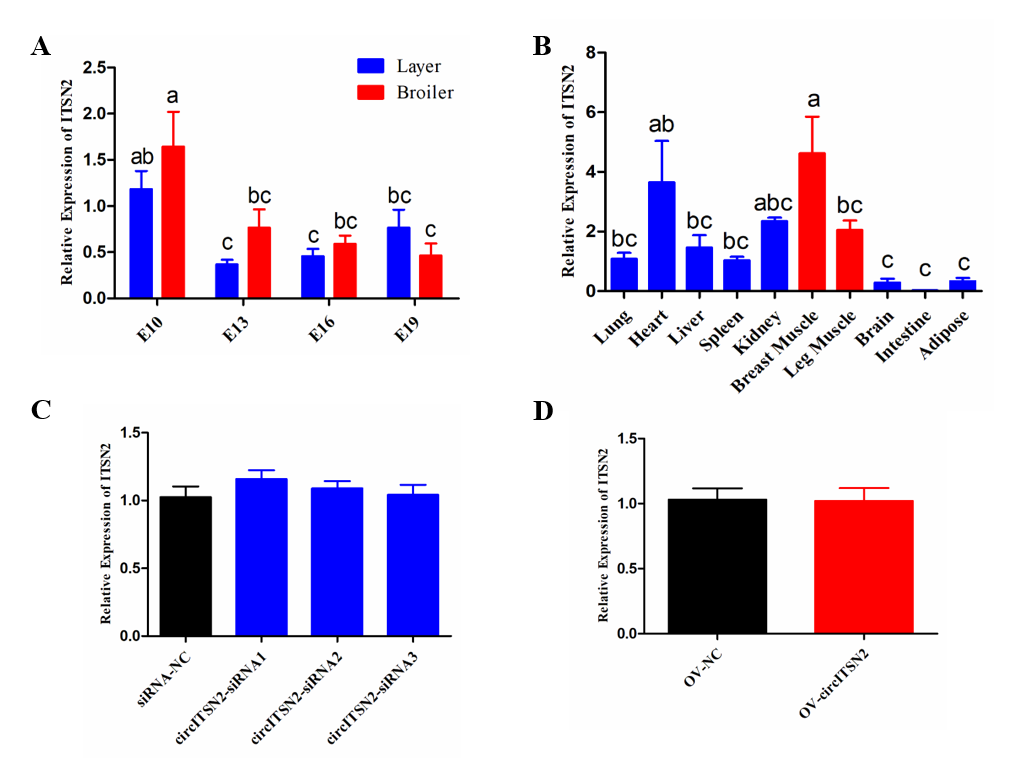


**Supplementary figure S1. (A)** The expression of ITSN2 in the muscle of two chicken lines with four different embryonic stages detected by qRT-PCR. **(B)** The expression pattern of ITSN2 in ten different chicken tissues. **(C)** The expression of ITSN2 in CPMs which transfected with unique siRNAs of circITSN2. **(D)** The expression of ITSN2 in CPMs which transfected with circITSN2 overexpression vector or empty vector. Data are presented as mean ± SEM for at least three individuals; ^ab^*P* < 0.05.


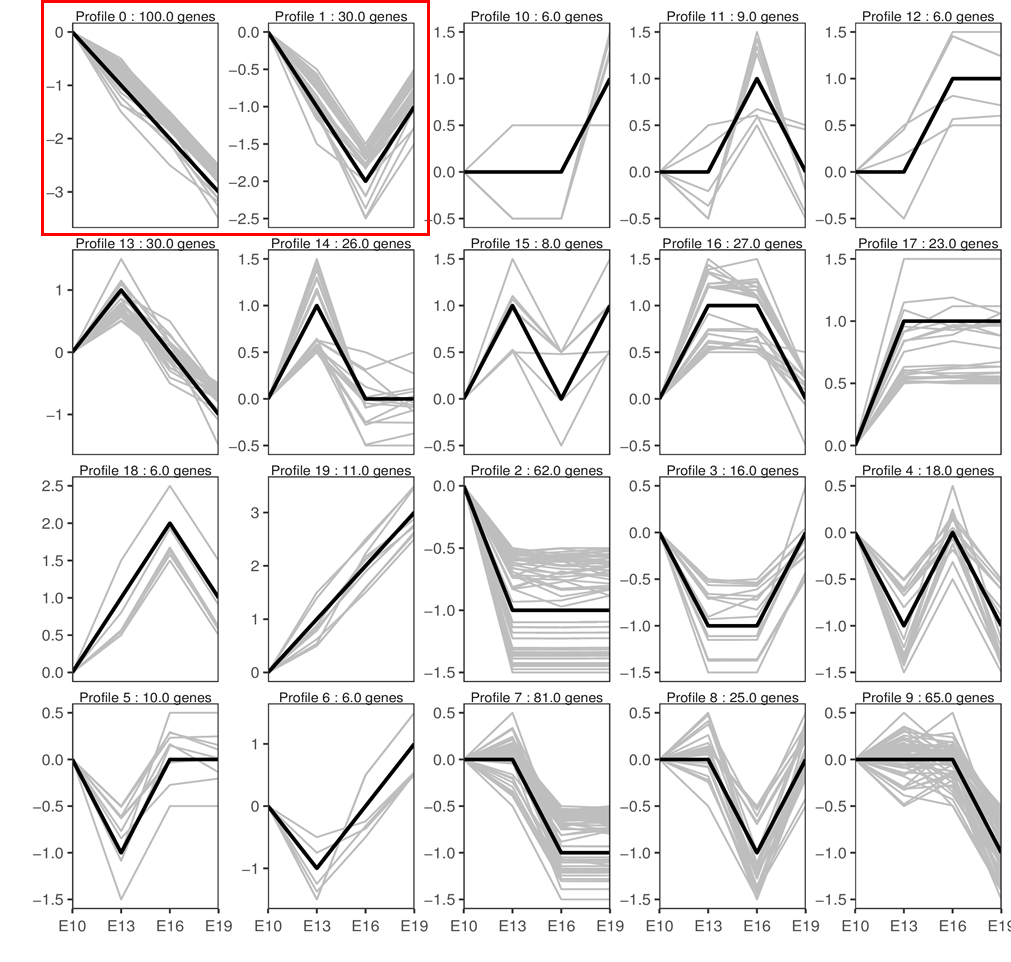


**Supplementary figure S2.** Expression trend analysis of miRNAs in the breast muscle of fast muscle growing broiler (FMGB) at embryonic day 10 (E10), E13, E16 and E19. The sequencing data is in SRA database with accession number PRJNA516545. The down-regulated miRNAs were selected in the red box.


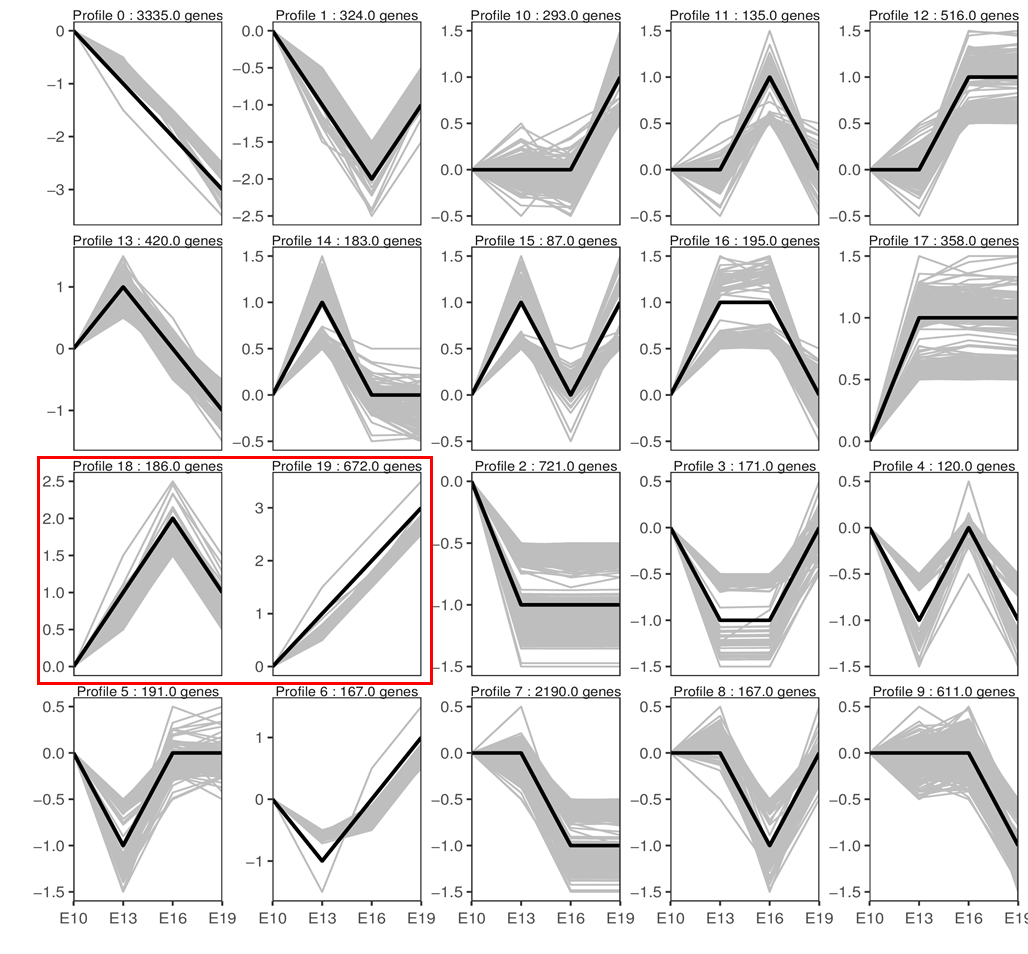


**Supplementary figure S3.** Expression trend analysis of genes in the breast muscle of FMGB at E10, E13, E16 and E19. The sequencing data is in SRA database with accession number PRJNA516545. The up-regulated genes were selected in the red box.
